# Multidrug-resistant *Klebsiella pneumoniae* ST70 harboring *bla*_*NDM*_ in a migratory Penguin

**DOI:** 10.1038/s41598-025-97816-4

**Published:** 2025-07-01

**Authors:** Sandryelle Merces Freire, Annelise Kyllar, Marina Côrtes, Thiago P. G. Chagas, Felipe Pinheiro, Renata F. A. Pereira, Fabio Aguiar-Alves, Bruno Penna

**Affiliations:** 1 Laboratório de Cocos Gram Positivos do Instituto Biomédico, Universidade Federal Fluminense, Niterói, Brazil; 2 Instituto de Medicina Tropical da Faculdade de Medicina, Universidade de São Paulo, São Paulo, Brazil; 3 Molecular Epidemiology and Biotechnology Laboratory, Faculty of Pharmacy, Universidade Federal Fluminense, Niterói, Brazil; 4 Pharmaceutical Sciences, Lloyd L. Gregory School of Pharmacy, Palm Beach Atlantic University, West Palm Beach, USA

**Keywords:** Clinical microbiology, Antimicrobial resistance, Bacterial genomics

## Abstract

The growing prevalence of antimicrobial resistance poses a global threat to human and animal health. In this study, we investigated the occurrence and genetic basis of antimicrobial resistance in a Magellanic Penguin (*Spheniscus magellanicus*) rescued off the coast of Rio de Janeiro, Brazil. The penguin presented a bacterial infection, identified as *Klebsiella pneumoniae*. Molecular analysis revealed the presence of several resistance genes, including those that confer resistance to carbapenems, beta-lactams, quinolones, and other classes of antibiotics. The bacterial strain belonged to Sequence Type 70 (ST70), a clone previously associated with human nosocomial infections. This study highlights the potential of migratory penguins as vectors of antimicrobial-resistant microorganisms, emphasizing the need for a One Health approach to address the complex interaction between environmental factors, animal health, and human well-being. The findings underscore the urgency of implementing strategies to mitigate the spread of multidrug-resistant bacteria in natural and urban environments.

## Introduction

The genetic determinants of antimicrobial resistance are naturally present in bacteria^1^. The frequent use of these drugs creates selective pressure that contributes to the emergence of resistant bacteria, threatening human and animal health^2,3^.

*Klebsiella pneumoniae* demonstrates a progressive increase in multidrug resistance and may be naturally present in the intestinal tract of animals. It is a main bacterial species responsible for nosocomial infections, with a high prevalence of multidrug-resistant strains^4,5^. Recent studies have reported the isolation of antimicrobial-resistant *K. pneumoniae* in wild animals^6,7^. The closer contact between wild animals and the urban environment highlights the importance of a one-health approach in controlling zoonosis, food safety, pollution management, and antimicrobial resistance control^8^. The human-animal interaction contributes to the exchange of genetic material between microorganisms and reinforces the dissemination of multiresistant bacteria. Understanding the importance of zoonotic transmission of antimicrobial-resistant bacterial strains can slow their spread and ensure human and animal health.

Cases of different species of wild animals carrying these microorganisms in their respective habitats have already been reported^9^. The marine ecosystem is constantly affected by wastewater runoff containing bacteria carrying antibiotic-resistant genes or the genes themselves^10^. Aquatic species can act as sentinels and vectors of multiresistant microorganisms^11,8^. In this context, penguins are migratory seabirds, possibly capable of carrying different types of microorganisms throughout their journey^9,12^. Migratory species like the Magellanic Penguin *(Spheniscus magellanicus*) are fundamental for conservation, as these provide important information about changes in the marine environment^13^. These penguins are endemic to South America, inhabiting mainly along the coast of Patagonia, Argentina, and Chile^14^. Although individuals of this species do not reproduce in Brazil, after the reproductive period (September to March), these individuals start their pelagic season (April to September), when adults and juveniles leave their colonies and migrate to the north along the coast of Uruguay and Brazil, searching abundant food resources, and to avoid the adverse environmental conditions found in winter in the region of their colonies^15^. During this period of the year, individuals are seen stranded along several Brazilian beaches, showing mainly signs of exhaustion, drowning, and infectious processes caused by several factors like overfishing, fisheries action items, oil pollution, and climate change, so some projects carry out the rescue of these individuals for veterinary care, rehabilitation, and subsequent release.

Thus, they have great potential to get infected and spread bacteria and antimicrobial resistance genes^9,16^. However, few studies relate the isolation of multiresistant bacteria in wild animals, particularly penguins. Therefore, we report a Magellanic-Penguin (*Spheniscus magellanicus)* case rescued on the Itacoatiara beach, located in the municipality of Niteroi, the coast of Rio de Janeiro, presenting a bacterial pneumoniae caused by *K. pneumoniae*.

## Materials and methods

A single Magellanic penguin was rescued on the beach of Itacoatiara, located in the municipality of Niterói - RJ, through the Santos Basin Beach Monitoring Project (PMP-BS). The sample collection was approved by the Animal Use Ethics Committee of Fluminense Federal University, and an authorization for the Capture, Collection, and Transport of Biological Material No. 755/2016 by the **Brazilian Institute of the Environment and Renewable Natural Resources** (Portuguese: *Instituto Brasileiro do Meio Ambiente e dos Recursos Naturais Renováveis*, **IBAMA**) for the RJ Area, under the coordination of Econservation Environmental Studies and Projects. All experiments were performed following relevant guidelines and regulations. Also, this study aligns with Animal Research: Reporting of In Vivo Experiments (ARRIVE - Animal Research: Reporting of In Vivo Experiments) guidelines.

The penguin presented symptoms of “Stranded Penguin Syndrome”, being evidenced by lean body score, hypothermia, marked dehydration, pale mucous membranes, and caseous formation in the oral cavity due to the presence of fish spines that pierced its mucosa, in addition to characteristic symptoms of drowning. After the first care and screening, the animal stayed in a Poultry Treatment Unit, where it received appropriate treatment with intravenous fluids, osmotic diuretic, glucose, and bronchodilator for stabilization and treatment of clinical signs presented. The penguin received a feeding protocol with fish porridge and vitamin supplementation until the beginning of solid feeding. Complementary tests demonstrated marked leukopenia, suggestive of an acute infectious process. After seven days of stabilization, a radiographic examination revealed changes representative of pneumonia. An endotracheal swab was collected for better evaluation and clinical management.

After 90 days of treatment, during which the penguin showed clinical improvement and without respiratory symptoms, the animal began to present high respiratory rates during auscultation and died on the 99th day. At necropsy, diffuse caseous masses were evidenced, with yellowish mucus content in the lung parenchyma, characteristic of an infectious process. In addition, the penguin also presented alterations characteristic of infectious pericarditis and hepatomegaly.

### Bacterial culture

A sample was collected by swabbing the tracheal region. The swab was transported to the Laboratory of Gram-Positive Cocos at Universidade Federal Fluminense and stored at 4ºC until processing. The sample was inoculated in TSB Broth (Tryptic Soy Broth) and incubated at 37ºC for 24 h. Next, after gram staining evaluation, the sample was inoculated onto Eosin Methylene Blue Agar (EMB), Mannitol Salt Agar, and Chromogenic Agar for KPC by the depletion technique. Blue-colored colonies, with a presumptive bright appearance of Klebsiella pneumoniae, were seeded on ATS Agar and incubated at 37 °C for 24 h. Identification was performed using the Matrix-Assisted Laser Desorption ionization-time of flight (MALDI–TOFms - Biotyper-Bruker) spectrometry method, as recommended by the manufacturer. A score ≥ 2000 was used for species-level identification.

### Antimicrobial susceptibility test

We evaluated the isolate’s susceptibility by disk diffusion method, using CLSI standard^17^. The antimicrobials tested were ampicillin, amoxicillin with clavulanic acid, cefazolin, ceftazidime, cefotaxime, cefoxitin, ceftriaxone, ceftiofur, imipenem, aztreonam, ciprofloxacin, norfloxacin, enrofloxacin, gentamicin, azithromycin, tetracycline, chloramphenicol, fosfomycin, nitrofurantoin, and sulfamethoxazole. The commercial Policimbac^®^ broth microdilution system determined Polymyxin B MIC (minimal inhibitory concentration). Resistance to three or more classes of antimicrobial agents defined the isolate as multi-drug resistant (MDR) according to the definition proposed by Magiorakos et al., 2012^18^.

### DNA extraction

Selected colonies were cultured on Tryptone Soy Agar (KASVI, Paraná, Brazil) and incubated aerobically at 37 ºC for 24 h. DNA was extracted using the Wizard Genomic DNA Kit (Promega, Madison, USA) according to the manufacturer’s instructions. The extracted DNA was quantified using the Quantus Fluorometer (Promega, Madison, EUA) according to the manufacturer’s instructions.

### Carbapenemase screening

Modified carbapenem inactivation test (mCIM) combined with EDTA-modified carbapenem inactivation test (eCIM) was applied for detecting the carbapenemase, followed by NG-Test CARBA 5 immunochromatographic assay. Additionally, multiplex polymerase chain reaction (PCR) was performed. Genes encoding the main carbapenemases (*bla*_NDM_, *bla*_KPC,_ and *bla*_OXA48_) were investigated using previously described primers^18^.

### Whole-genome sequencing and genome assembly

Library preparation was performed using Illumina DNA prep (M) Tagmentation (96 samples) and Nextera DNA CD index (96 indexes, 96 samples) (Illumina, San Diego, California, USA), according to the manufacturer’s instructions. Libraries were subsequently quantified using Qubit and the dsDNA HS-kit (Thermo, Massachusetts, USA). Finally, the sample was loaded on a HiSeq2500 system and ran for 201 cycles (PE125), pair-end (500 bp library) using HiSeq Rapid SBS Kit v2 chemistry. The reads obtained were trimmed using BBDuk with default parameters. The assembly was performed with *de novo* and reference assembly protocols using Velvet 1.2.10 ^42^ and Geneious 2022.0.2 (Biomatters, Auckland, New Zealand), using genome 6109 of *Klebsiella pneumoniae* as reference (CP051149).

Genome annotation was performed at the Pathosystems Resource Integration Center (PATRIC 3.6.12). MLST typing was performed in silico using the MLST tool from the Center for Genomic Epidemiology (CGE) (https://cge.food.dtu.dk/services/MLST/*).* The genetic determinants of resistance were searched using two tools: ResFinder from CGE (https://cge.food.dtu.dk/services/ResFinder/*)* and The Comprehensive Antibiotic Resistance Database – CARD (https://card.mcmaster.ca/home*).* The genetic determinants of virulence were analyzed using VirulenceFinder (CGE). The visualization of plasmid alignments was assessed with EasyFig v2.2.5. One hundred forty-three publicly available genomes were included in the phylogenetic tree reconstruction. The pathogen watch database was used to download 85 ST70 genomes, mostly isolated from humans, and the NCBI database to download 58 genomes isolated from birds and the environment (supplementary table). The sequences were aligned using bowtie2. Multiple sequence alignments were reconstructed, from which phylogenetic trees were inferred using PhyML on the REALPHY pipeline.

## Results

All identifications confirmed that the isolated bacteria were *Klebsiella pneumoniae*. Regarding susceptibility to antimicrobials, it resisted all antibiotics tested except for Polymyxin B (MIC = 1 µg/mL). The *K. pneumoniae* isolate showed intermediate sensitivity to norfloxacin and enrofloxacin, while the mCIM/eCIM tests and the immunochromatographic assay suggested a metallo-carbapenemase production. The multiplex PCR assay was *bla*_NDM_-positive. No other carbapenemase gene was detected.

**Fig. 1:**
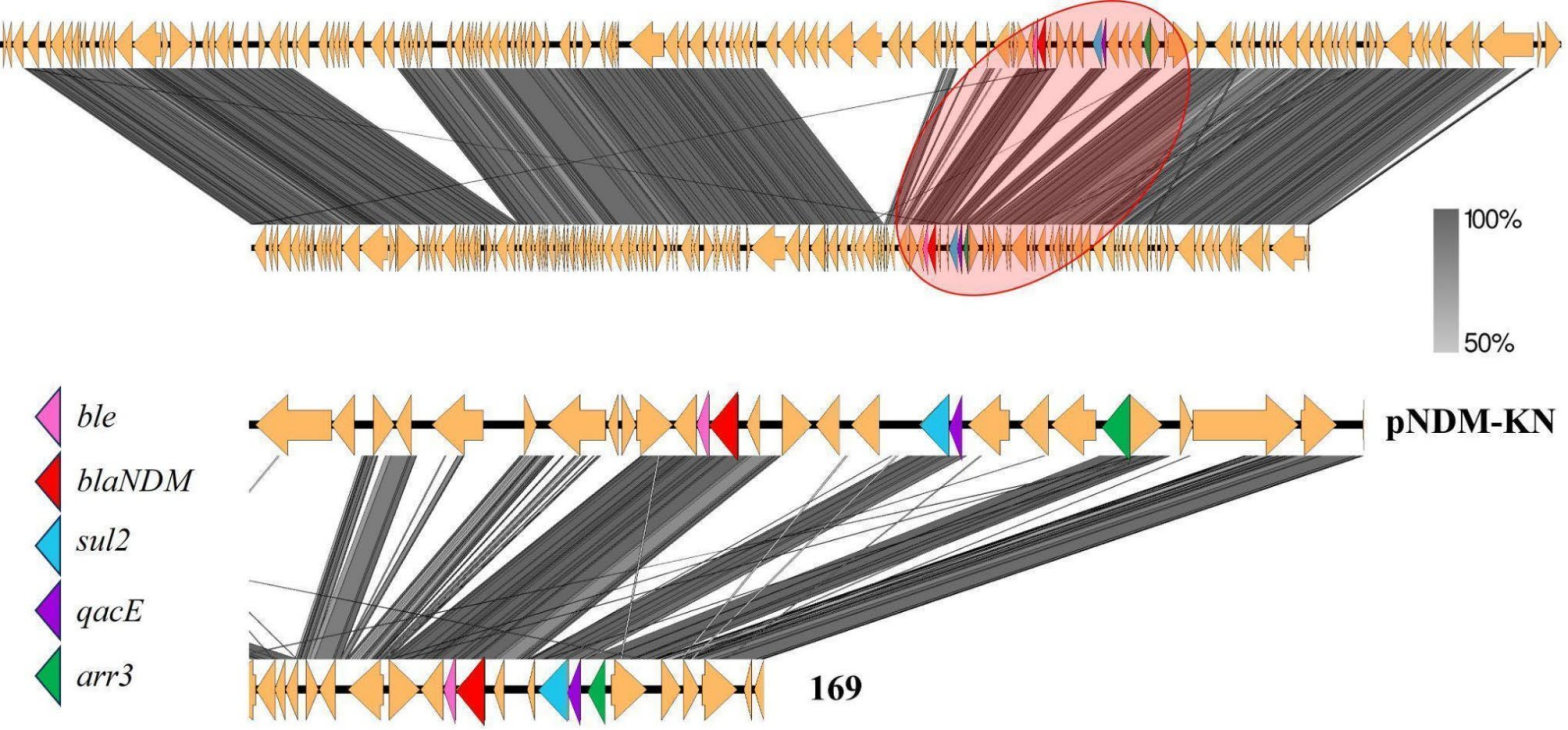
Alignment of the plasmid pNDM-KN (accession number JN157804) with the plasmid carried by the penguin isolate from this study. Below: Zoom of the region highlighted by the red circle. Resistance genes are shown in colors.

**Fig. 2:**
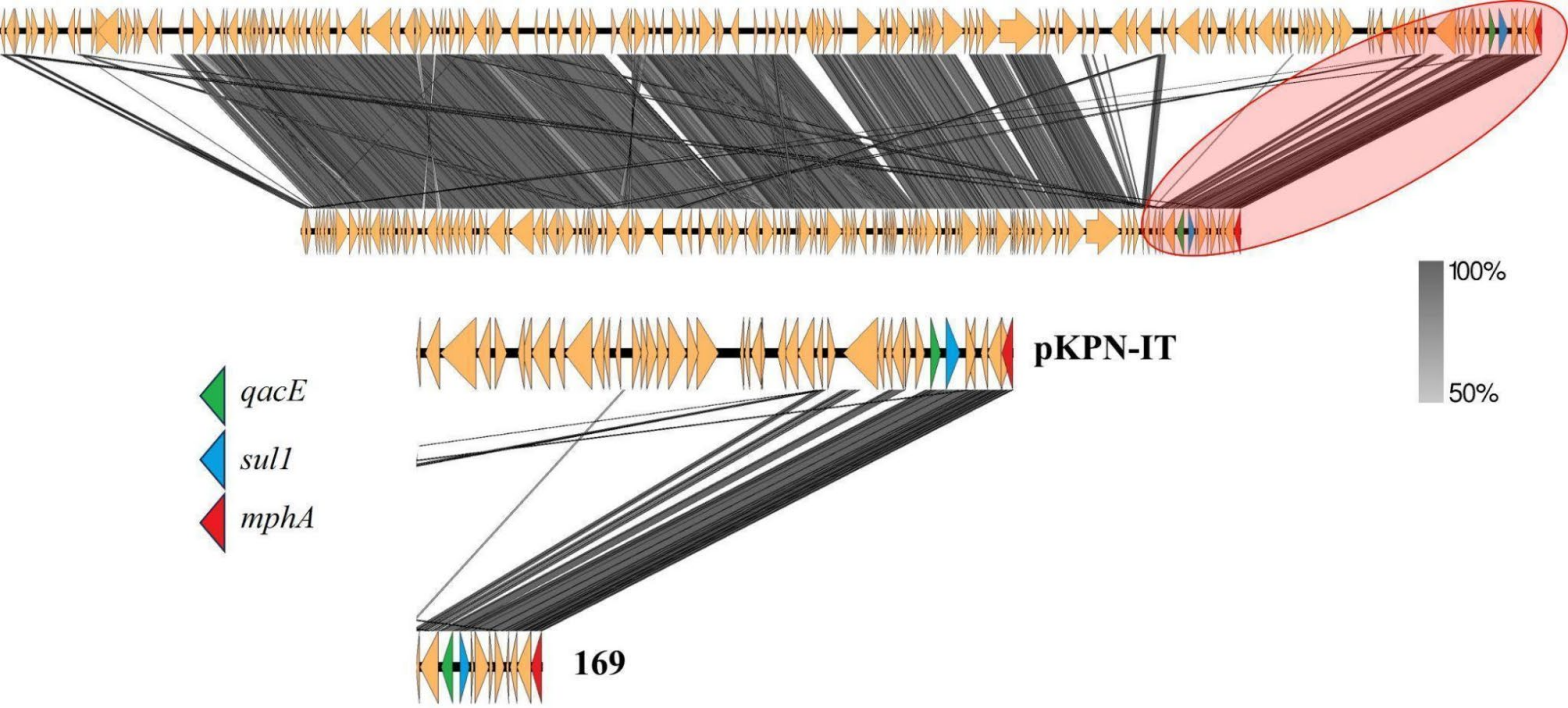
Alignment of the plasmid pKPN-IT (accession number JN233704.1) with the plasmid carried by the penguin isolate from this study. Below: Zoom of the region highlighted by the red circle. Resistance genes are shown in colors.

**Fig. 3:**
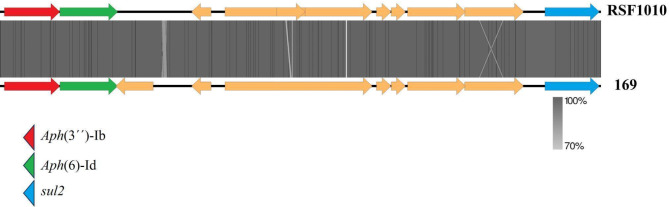
Alignment of the plasmid RSF1010 (accession number NC_001740) with the plasmid carried by the isolate 169 from this study. Resistance genes are highlighted in colors.

Sequencing identified *K. pneumoniae* subsp. *pneumoniae* with resistance genes for quinolones (*oqxA*; *oqxB*; *qnrB1*; *aac*(6)-Ib-cr_1), macrolides (*mph*(A); *erm*(B)), beta-lactams (*bla*CTX-M-15; *bla*OXA-1, *bla*SHV-32, *bla*TEM-1 C, *blaNDM*-1), chloramphenicol (*floR*, *catB*3), sulfonamides (*sul2*; *sul1*), rifampicin (ARR–3), aminoglycosides (*aac*(3)-IIa, *aph*(3’)-VIa, *aph*(3’’)-Ib, *aph*(6)-Id), streptogramins and lincosamides (*erm*(B)), fosfomycin (*fosA*), tetracycline (tetA), trimethoprim (dfrA14). Many of these genes were carried by plasmids, as shown in Figs. 1 and 2, and 3. The genes *ble*, *blaNDM*, *sul*, and *quac* are carried on a plasmid similar to pNDM-KN (accession number JN157804; Fig. 1), an IncA/C plasmid. The genes *quac*, *sul*, and *mph* are carried on a plasmid similar to pKPN-IT (accession number JN233704.1; Fig. 2). The genes *aph(3)*, *aph*(6), and *sul* are carried on a plasmid similar to RSF1010 (accession number NC_001740; Fig. 3). Additionally, the studied isolate also presented virulence genes codifying for fimbriae (*fimH* and *mrkA*), siderophore receptor (*fyuA*), siderophore yersiniabactin (*irp2*), ferric aerobactin receptor (*iutA*) and complement resistance (*traT*).

**Fig. 4:**
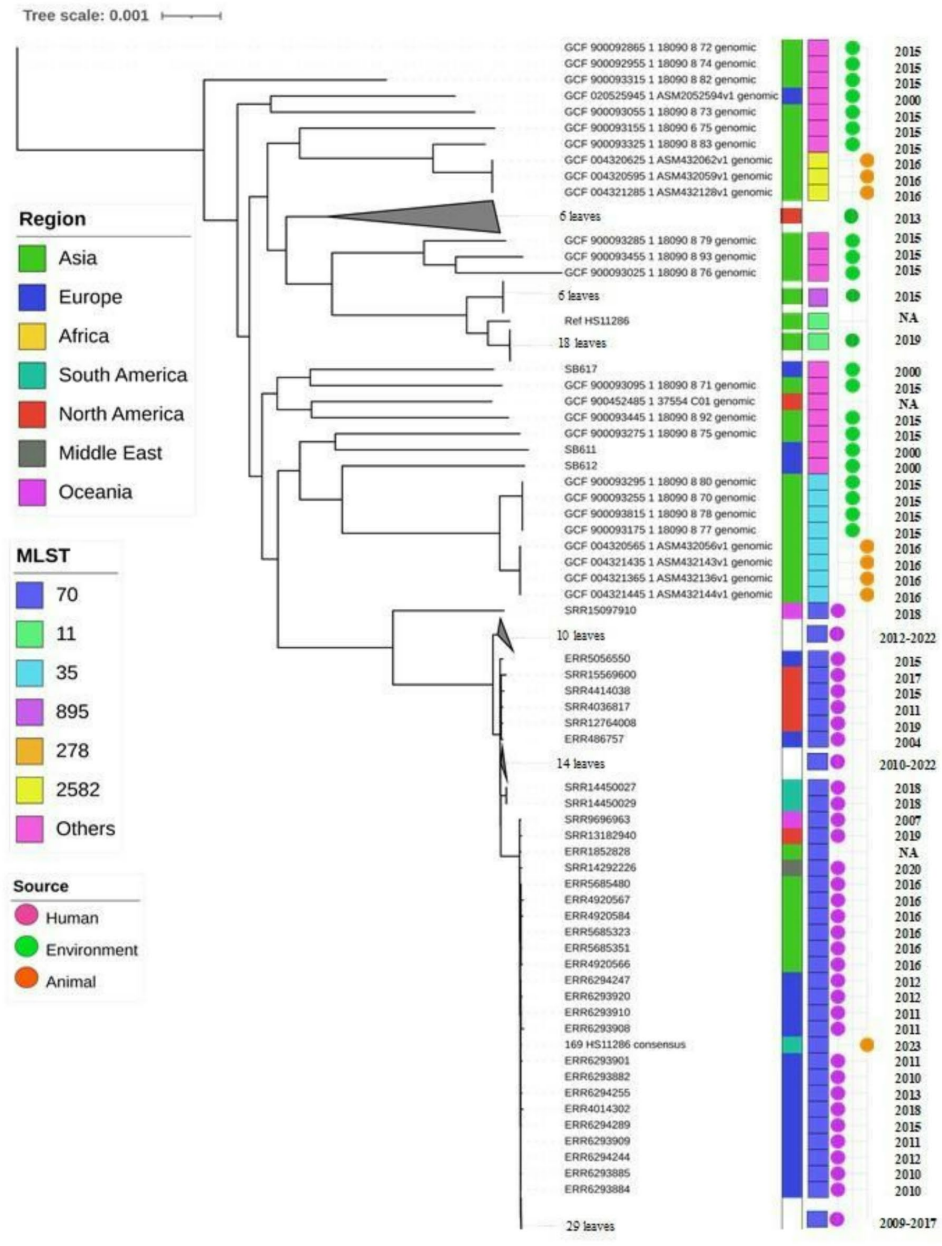
Maximum likelihood phylogenetic tree of 169 (in bold) with 143 *klebsiella pneumoniae* genomes publicly available at NCBI and pathogen watch. Colors are shown in the legends. Not available information is in black.

MLST identified the bacterium as being from ST70. The phylogenetic tree showed that the sample branched together with other clinical isolates belonging to ST70, mainly those isolated in Europe (Fig. 4).

## Discussion

The rise in antimicrobial resistance among bacterial pathogens represents a vital concern, and the role of wildlife in disseminating these resistant strains is becoming increasingly recognized. Our study unveils a multidrug-resistant *Klebsiella pneumoniae* in a Magellanic Penguin, a migratory seabird, implicating these animals as potential vectors for antimicrobial-resistant microorganisms. Notably, this study demonstrates the interconnection between environmental pollution, human-animal interactions, and the emergence of resistance.

Reports of isolation of resistant bacteria in wild animals are progressively increasing^19,20^. Analogously to this study, research carried out in wild hedgehogs highlighted *Klebsiella* spp. as the genus with the highest proportion of resistance genes^21^. A study in Senegal detected different clones of *K. pneumoniae* in wild chimpanzees and termites^22^. That same study reports the identification of clones harboring plasmids that carried the *blaCTX-M-15* and *blaOXA-1* genes, as well as the genes that confer resistance to aminoglycosides [*aac*(3)-Iia and *aac*(6’)Ib-cr], quinolone (*qnrB1*), sulfonamides (*sul2*) and chloramphenicol (*floR*), similar to what was found in this research. One of the resistance genes found in *K. pneumoniae* isolated in the present study was the *blaNDM-1* gene, which refers to “New Delhi Metallo beta-lactamase” and confers resistance to broad-spectrum antibiotics such as carbapenems. Ahlstrom^23^ isolated enterobacteria resistant to carbapenems from wild birds in different world regions. *K. pneumoniae* carbapenemase (KPC) presenting the *blaNDM-1* gene was isolated among these bacteria. Another study detected the presence of *Escherichia coli* bacteria carrying the *blaNDM-1* gene in a penguin on the Brazilian coast^24^. Although there are few reports of the isolation of multidrug-resistant *K. pneumoniae* in penguins, previous studies reveal its appearance in wild animals, especially when dealing with migratory birds^16,25^.

The CTX-M group, which confers resistance to beta-lactams, is recognized as the most prevalent variant of Extended Spectrum Beta-Lactamase (ESBL), with the CTX-M-15 gene being particularly notable in bacteria found in both wild animals and humans^9^. In Brazil, researchers isolated *K. pneumoniae* containing *blaNDM-1* and *blaCTX-M* genes from hospitalized patients^26^. The present work reports, for the first time, the occurrence of *K. pneumoniae* that carries *CTX-M-15* isolated from a free-living penguin. Thus, identifying these resistance genes in the urban and wild environment reveals the danger of the emergence of multidrug-resistant bacteria due to the excessive use of antimicrobials and consequent dissemination through migratory animals.

In this study, multilocus sequence typing identified *K. pneumoniae* ST70, a clone previously reported in nosocomial infections in humans in Brazil^27,28^ and India^29^. A recent study revealed the presence of this clone in a blue and yellow macaw (*Ara ararauna*) in the central-west region of Brazil^30^. This ST70 is more associated with nosocomial infections in humans. Other studies with different species of penguins found several STs differing from our results. The isolation of *K. pneumoniae* ST70 in distant continents and different species is alarming worldwide. When we evaluated studies that conducted WGS analyses, this was the first study that assessed the ST70 from animal origin in South America. Since this ST has already been reported in Hospitals in Rio de Janeiro^27^, one possible explantion is that the penguin got in contact with bacteria from hospital waste.

In conclusion, our study highlights the emergence of multidrug-resistant *Klebsiella pneumoniae* in migratory penguins, underscoring the importance of a One-Health approach to combating antimicrobial resistance. Collective efforts are required to mitigate the spread of antimicrobial-resistant microorganisms, encompassing environmental conservation, responsible antimicrobial use, and collaborative research across disciplines. By addressing these challenges, we can safeguard both animal and human health in the face of the escalating threat of antimicrobial resistance.

## Data Availability

Sequence data that support the findings of this study have been deposited in the Gene bank with the primary accession code JBDLPA000000000; BioSample SAMN41189574.
